# Abstracts and Gleanings

**Published:** 1887-07

**Authors:** 


### ABSTRACTS AND GLEANINGS.

**Placenta Prævia, with Uterine Hemorrhage.**—Case 1. Was called June 20th to see Mrs. S., in consultation with the family physician. Upon arrival there I was met at the front gate by the doctor, who hastily and excitedly informed me that the patient was in labor; that it was a case of placenta prævia, and the patient was flooding. He had given ergot, and used the tampon, but the means were doing no good. He wound up by asking what I did in such cases. I answered by saying that I would first arrest the hemorrhage, and then do what was to be done. I removed the tampon, found the os dilated about two inches; the edge of the placenta rested across the edge of the cervix, but the larger portion of it lay to the left and posteriorly. We removed the pillows from under her head, and raised the foot of the bed high enough to slip a chair under the foot of each bed-post, making the foot eighteen or twenty inches higher than the head. The hemorrhage casèd at once, and we had nothing to do but sit around and wait for dilatation of the os uteri.

Inquiry into previous history revealed the fact that she had hemorrhage about two months previously, and at three different times afterwards. Having waited about two hours, during which time the pains were strong, and the os now being well dilated, we ruptured the membranes, pushed the edge of the placenta over to the left and back, and held it there until the child's head entered the os. In a short time afterwards the child was born, and the patient made a good recovery.

Case 2. I was called December 4th in consultation to see Mrs. G., four miles in the country—found her flooding. She lacked six weeks of her full term of utero-gestation No pains—not in labor. We raised the foot of the bed as in the preceding case, and the hemorrhage ceased immediately. Before leaving we directed the foot of the bed to be let down in one hour, and in case the hemorrhage returned to again elevate it. And if the flooding became too troublesome, we announced that we might have to produce premature delivery. There were several recurrences of hemorrhage, but all were soon arrested.

I was recalled January 12th; found patient in labor; os dilated to the diameter of two inches; foot of bed had been raised, and yet there was some wasting at each pain. I found the placenta across the cervix, but the larger portion extended posteriorly. We again raised the foot of the bed to about twenty-two inches, then called for cotton batting (not rags) and half a teacupful of lard. We wadded suitably sized pieces of the textile to be introduced without giving too much pain. We smeared them with the lard, and plugged the vagina; seven or eight pledgets were used in the tamponing process.

The hemorrhage now being arrested, and pains tolerably strong, we waited for dilatation. In about one hour and a half, the pains becoming quite strong, we removed the tampon; found the os well dilated; ruptured the membranes, and pushed the edge of the placenta back into the uterus. The head now presented, and the child was born in a few minutes. Both mother and child did well.

In cases of central or complete placenta prævia, the danger to the child is greater, but not more so to the mother. I am of the opinion that such cases are not as common as generally supposed. The two cases reported were thought to be complete, yet were only partial. When complete placenta prævia does occur, we are to arrest the hemorrhage until the os is dilated, then at once deliver; carefully, completely, and rapidly detaching the whole of the placenta, and bringing it away. Since the hemorrhage proceeds from the uterine surfaces, and not from the placenta, there need be no fear of further loss of blood; for the open sinuses are occluded by the child's head. If there is not too much exhaustion, my usual plan of delivery is to pass the hand along the side of the placenta, detaching as I proceeded, and selecting, if possible, the side least attached. As there is no rule of procedure that can be laid down in all cases, we must be guided by the particular conditions existing.

Case 3. Was called in consultation to see Mrs. L., Sept. 28th, several miles in the country. Arrived at 11 p. m., and learned from the attending physician that the patient had been delivered at six in the evening, and that hemorrhage had continued ever since. He could not remove placenta, had given ergot, and finished up by saying he had tried my plan of raising the foot of the bed, but all had failed. On looking around I observed the foot of the bed to be raised about three or four inches, and yet he called it my plan. We now raised it about twenty or twenty-two inches high; the hemorrhage ceased immediately, and though the patient was pulseless and much exhausted, I introduced my hand into the uterus, commenced at the upper portion of the placenta, and detached it—keeping my fingers firmly and closely to the walls of the uterus in pealing it off, and being careful not to leave anything adhering to the inside of the uterus. I remove all with ease, and without the loss of much blood. The patient remained quite feeble for three or four days, but rallied well in the end.

In cases of this kind I lay down the following rules: Raise the foot of the bed from eighteen to twenty-four inches. Then use a tampon previous to the expulsion of the fœtus. If the above instructions be carried out, I do not see how it is possible for any woman to die of hemorrhage. Plethoric patients may waste for a short time, and that, too, without detriment. There will be enough blood retained in the body to sustain life, and to make a good recovery.—*Eclectic Medical Journal.*

**Charcoal and Camphor.**—A mixture of equal parts of camphor and animal charcoal is recommended by Barbocci for preventing the offensive odor and removing the pain of old excavated ulcers. The camphor is stated to act as a disinfectant, and the charcoal absorbs the offensive odors.—*British Medical Journal.*

**Suppurative Inflammation of the Middle Ear.**—In cases where the perforation is recent, Dr. Savage, of Nashville, (*Nashville Medical Journal*) suggests the following new plan of treatment:

My new plan of treatment, which I propose to make known in this article, is most suitable to cases in which the perforation is recent, as well as those cases in which an artificial opening has just been made. I feel that I am not far from the truth when I say by my plan most patients can be cured in one day at one sitting, and that the cases will be rare that will require more than from three to five sittings.

Simple as is the plan, it is as follows: By Politerization blow all the pus possible out of the drum cavity through the small opening in the drum membrane into the auditory canal, whence you can easily remove it by means of absorbent cotton on a probe or angular forceps; warm a solution of nitrate of silver (gr. ss to j) in water (ʒ j), and, having had the patient incline his head toward the well side, fill the auditory canal half full of the solution; *then ask the patient to expire fully, to close his mouth and hold his nose, and then make an attempt at respiration.* Since air cannot enter through either nose or mouth the result of the attempt to inspire is exhaustion, more or less complete, of all cavities communicating with the nose and naso-pharynx. The diseased drum being one of these, it must necessarily become exhausted. As a consequence, the drum membrane is drawn in and its perforation made more open, so that through it the solution in the auditory canal is either drawn into the drum cavity by suction or forced into it by atmospheric pressure, filling it more or less completely. During this attempt at inspiration both patient and doctor can hear the noise made by the rushing of the fluid into the drum cavity. The patient can now elevate his head, and the doctor can remove, by means of absorbent cotton, the fluid remaining in the auditory canal.

After a minute, the fluid which has been drawn into the drum must be blown out by Politzerization, and removed from the canal just as the pus was in the first instance. After three to five minutes, the same process should be gone through with again, and then again, after a like interval, until the medicine has been made to enter the drum cavity from three to five times at the one sitting. One minute after the last suction, the medicine must be blown out as already indicated, and the auditory canal dried.

Your patient is apt to return to the office on the morrow, with the statement that there has been no return of the discharge, and that he has been comfortable in every respect since the treatment. If, however, he should state that pus has reformed, then you can not do better than repeat the procedure of yesterday. In most cases a cure will result from the first treatment; in all cases a cure will result more rapidly than from any other plan of treatment.

The inflammation having been cured thus early, the edges of the incision or the margins of the perforation grow together, and within a few days the hearing distance is normal.

Having devised this plan of treatment on the 19th day of last

June, I have had the opportunity of resorting to it only a few times in recent cases, all of whom recovered from the one treatment, at the one sitting. In one case, however, there was a renewal of the discharge at the end of one week, on account of a new cold, at which time I gave him one more treatment, resulting in a cure again. One month later I saw him, and there had been no return of the discharge. The perforation had closed completely, and his hearing distance had extended to the normal.

As compared with other plans of treatment, mine has the advantage in simplicity of application, in adaptibility to a greater number of patients, children included, in a rapidity of cure, and a consequent restoration of hearing to, or near to, the normal, and in pleasantness to patients.

**The "Rational Method" of After-treatment of Cataract Operations, Iridectomies, etc.**—This method consists in the rejection of bandages, compresses, and the dark room, and the substitution therefor of a strip of isinglass plaster applied to the lids and an ordinarily lighted room, the other conditions as to rest, etc., remaining as before, or according to the individual judgment or habits of the surgeon. These seem extremely radical changes of the routine treatment laid down by von Grafe, and, with the few exceptions to be noted, universally followed by the profession. The advantages claimed for it by its supporters may be briefly classified as follows:

1. General. No special room is required; if in a private house, no artificial darkening; if in a hospital, the common wards may be used. Better ventilation, sanitary surroundings, and comfort are secured. The patient's friends or nurse may be with him in more cheerful conditions, may read to him without weak artificial light, etc. The long and dreaded period of darkness is obviated, the discomfort of a close bandage over the eye is avoided, the inflammatory weakening and unhealthy effect of this padding and warmth to the lids and adjacent tissues done away with.

2. The examination of the eye is easy and thorough, and an earlier discovery of bad results is secured. This is chiefly because the eye, being in receipt of its natural stimulus all the time, does not become photophobic, and a more frequent and accurately thorough examination is at all times possible. Incipient inflammation, œdema of lids, etc., is detected, the secretions, such as tears, discharges, etc., are not pent up in the already suffering eye, but have at all times a natural and ready drainage through the not rigidly joined palpebral fissure. The introduction of mydriatic, myotic, or antiseptic medicaments is at all times easy, without any disturbance of dressings.

3. Dangerous complications and sequelæ are evaded. Even the lightest; most uniform, and carefully applied bandage must exercise a certain pressure upon the front of the globe; it would not be a bandage did it not do so; and a body as soft and pliable as the globe must be somewhat flattened, regularly or irregularly, as the case may be. But however little this pressure, a symmetry of the cornea must result, and with the inevitable result that coaptation of the lips of the wound is not perfect. Hence, two serious and persistent evils—prolapse and entanglement of the iris in the wound, and, after recovery, astigmatism—are avoided.

4. The period of confinement is shortened, and, from the absence of photophobia (assiduously *cultivated* by the old method), the duration and comfort of that convalescence is rendered more desirable; whilst the tests and application of spectacles are sooner reached, followed by the earlier resumption of the ordinary habits of life.

The practical application and introduction of the method to the profession are due to Dr. Charles E. Michel, who sets forth his reasons for adopting it, and the results of twelve or fifteen years of practice, in *Knapp and Schweigger's Archives* for September. From Michel, Dr. Chisolm, of Baltimore, learned the method, and, adopting it enthusiastically, reports his success in *The Maryland Medical Journal* of June 19th, 1886, and in the June number of *The American Journal of Ophthalmology.* Lastly, Dr. Simeon Snell, of Sheffield, England, being struck with the reasonableness of the plan as described by Dr. Chisolm, adopted it in his own practice, and bears witness to its value in *The Lancet* for September 18, 1886.

Dr. Michel gives no definite statistics of his cases, but says that, "as the results were so uniformerly satisfactory from the first, I have never deemed it worth the time and labor of making out a statistical statement for comparison." Dr. Chisolm states that the results of twenty-four cataract operations, eight iridectomies and seven capsulotomies were most gratifying, and brilliant illustrations of this simple treatment." Snell reports eighteen cases in hospital and private practice, and, quoting Chisolm, endorses these words: "The revolution in the after-treatment of cataract and iridectomy patients in this hospital is complete. From this time forth, all bandages, compresses, and dark rooms will be among the things of the past, to be remembered only for the discomfort they occasioned." As to the plaster, each operator uses a different size and shape, Michel wisely urging that a large one communicates the motions of the face in chewing, speaking, etc., to the eye. He uses a strip of gold-beater's skin, one-half inch wide by one and a quarter inches long, over the palpebral fissures, leaving the latter loosely joined.

Prof. Thomson has banished dark rooms and thick compresses from the Ophthalmological Department of Jefferson Medical College Hospital since its opening in 1887. The patients are placed immediately after an operation, done with antiseptic precautions, in the open wards of a general hospital; the soft texture of a black knit, wool or silk bandage, with one thickness of patent lint placed over the eyes, is all the protection given. The above method has proven so satisfactory, both to the personal comfort of the patient and visual recovery, that at a future time full statistics of his cases will be published. We may mention that in the last series of one hundred cases, but two can be placed upon record as total losses, one by panophthalmitis the other by iridocyclitis which may yet be benefited by a future operation.

It will be seen that the "revolution" is based upon the fundamental axioms that light is the natural stimulus of the eye, and that the lids are its natural splints. So, far, therefore, as the application of these principles is consistent with the absolute demand for rest, the method seems to deserve its chosen epithet, "rational."—*American Journal of Medical Sciences.*

**Cancers—Orders and Treatment.**—Reply to Dr. P. C. Smith, page 56, *Brief* for February, as to a specific for cancer. Doctor, I will state, after many years' experience in the treatment of cancers, that there is no specific yet known. There are, however, remedies to which the cancerous growth will yield as readily as fevers, pneumonia, or any of the common diseases, to the proper remedial agents that are known to relieve the diseases for which they are given. Now, in treating cancers, as in all other diseases, we must be able to correctly diagnose the case. Just as well treat all fevers with the same remedies, as to try to treat all cancers with the same remedy. If we can determine to which order a given case belongs, we are then in a position to prescribe the remedies that will be indicated, and not grope along in the dark.

There are five orders of cancers: scirrhus, medullary, epithelial, colloid, and melanotic cancers—each of these orders have varieties. I will now give my treatment of the different orders:

For scirrhus, I use the following, which has given me better satisfaction than any other—and I have tried a great many:

℞. Acid. Arsen.................................ʒ i,

Ext. Phytolac. Dec.............................ʒ ii,

Ext. hyoscyam..................................ʒ ii.

M. Ft. salve: Apply to cancer q. s. to cover it.

Allow it to remain twenty-four or thirty hours, remove it and dress with vaseline; in a few days, the abnormal part will drop out. After the inflammation passes off, if any induration is left apply acid nitrate mercury, which will cause the part to drop out the next day.

For medullary cancer, I find the following the best:

℞. Pulv. galangal rad...........................ʒ i,

Pulv. sanguinariæ rad..........................ʒ i,

Zinci chloridi q. s. to make paste.

M. Sig. Apply as above.

If cancer is not all removed, apply same, and dress with olive oil.

For epithelial:

R. Acid. arsen..................................ʒ i,

Pulv. sanguinariæ..............................ʒ ii,

Mucillag. acaciæ q. s. to make paste.

M. Sig. Apply as above.

Dress with powd. ulmus till parts drop out, then with vaseline.

For colloid:

℞. Zinci chloridi . . . . . . . . . . . . . . . . . . . . . . . . . . . . . . . ℥ ss,

Auri chloridi . . . . . . . . . . . . . . . . . . . . . . . . . . . . . . . ℈ ss,

Pulv. galangal rad . . . . . . . . . . . . . . . . . . . . . . . . . . . ʒ ii,

M. Sig. Make paste, and apply as in the other cases; if hardness remains, reapply same; dress with vaseline.

Encourage patients to eat anything that will digest readily. Give iron in same. I use Vallett's mass, also syr. ferri iodidi.

The melanotic variety I have never treated. Have seen only one case.

These are the curative remedies that I now use. Of course, there are many circumstances that would suggest treatment other than I have mentioned. All the indications must be met by the physician as they arise, as in other diseases. Use no water on the sore from first to last. Be careful in dressing, so as not to wound the tender granulation. Sometimes abnormal granulation will be seen springing up from one edge, or from the center. In all cases where this occurs, the acid nitrate mercury, with a little muriate cocaine added, will remove the growth without pain. Try touching it with a glss brush; do not allow the sore to heal until all hardness has been removed. Some cases require several applications.

I have treated several hundred cases in the last fifteen years, with a success of about fifty per cent. The osteoid variety, I am of the opinion, cannot be successfully treated; but the orders I have mentioned are, I know, amenable to treatment. The only difficulty is in diagnosing the case; if the diagnosis is correct, we can treat the case with confidence.—*Dr. H. Wilson in Medical Brief.*

**Sciatica Treated by Galvanism.**—In *The Lancet*, July, 1886, Dr. W. E. Stevenson gives a short account of sixty cases of sciatica treated by means of galvanism. In *The Lancet*, July 19, 1884, the author published a paper upon the subject in which he described the mode of applying the current. Out of the sixty cases, thirty-seven were cured; eleven improved only; nine did not remain under treatment long enough for a certain result to be noted; two were still under treatment, and one had a return of sciatica, which was cured a second time. The number of applications needed to produce a cure was about six to eight; some few patients came twelve or fifteen times. The author concludes by stating that the favorable results obtained by him bear out the opinion given by Dr. Stone in his Lumleian lectures.—*Medical and Surgical Reporter.*

This record comes to us from across the Atlantic. If the reader will turn to the October, 1885 number of this journal he will find a record of a most obstinate case of sciatica. The whole of the great sciatica with its branches having been implicated (the most excruciating pain being at the point of emergence of the nerve from the cavity of the pelvis), the relief of which was finally accomplished, after four weeks, by twice or thrice daily repeated and prolonged galvanizations with a strong twenty-four-cell descending galvanic current and sponge electrodes.

He will also there find incidental mention of another and equally intractable form of neuralgia, a case in which all branches of the seventh pair were implicated, the convulsive paroxysms of pain recurring incessantly, which yielded to galvanism after having been treated by other means for nine years.

The cases were reported for another purpose, namely, to record some cases of temporary mental aberration following the cure of prolonged neuralgia; but they serve to show the value of a therapeutic practice older than Meyer, Benedict or Anstie, and so common in successful neurotherapy as to no longer require special testimony in confirmation.

To treat a persistent, grave neuralgia, without galvanism, is to neglect the most valuable and certain of all permanent resources, not even excepting the knife, for a cut nerve may reunite or collateral branches show the often remaining central pain.

In all neuralgias of nerves which can be traversed from center to periphery by the galvanic current skillfully regulated galvanism is the best and most certain and permanent of all therapeutic resources. Its therapeutic value needs no further confirmation.—*Neurologist.*

**Distilled Water in Eye Lotions.**—Physicians and pharmacists generally assume that distilled water is the best means of dissolving or diluting drugs, and in the great majority of cases this is undoubtedly the case. As a rule, the solvent should be absolutely pure and neutral in itself; but in some instances this very neutrality of the solvent may interfere with the intended action of the substance dissolved. A curious illustration of this exception to the rule was given sometime ago by an eminent English oculist in a communication to a London journal. Our readers know that an aqueous solution of boracic acid is one of the most useful of eye lotions; but we remember, that as prescribed for the writer several years ago by one of the best oculists in this city, the order was for "10 grains in *distilled water.*" Our English expert, however, says he has found that, when the lotion is made of distilled water, its use is attended with some discomfort and smarting; and, what is more singular, the irritation is even greater when there is no boracic acid in solution. "Anybody," he says "can verify for himself that a drop of distilled water in the eye seems to be a most unpleasant and foreign element."

The simple explanation of this is, that well or spring water contains salts in solution which make it slightly alkaline, thus rendering it somewhat more neutral to the *conjunctiva*, a tissue ordinarily bathed in the lachrymal secretion, which contains about one per cent. of solids, chiefly chloride of sodium, or common salt. The addition of two grains and a half of this salt to the ounce of distilled water renders any lotion for the eye more soothing and more beneficial. This, our friend says, he has verified by experience; and we commend the fact to the attention of oculists and others interested.—*Popular Science News.*

**The Treatment of Acute Intestinal Obstruction.**—In the *South-Western Medical Gazette*, Dr. L. S. McMurty, of Danville, Kentucky, discusses at length the subject of acute intestinal obstruction. Coming to treatment, he believes the most common error is the administration of purgatives and irritating enemata with the view of forcing a passage through the bowels. To use purgatives to overcome internal strangulation is as unreasonable as to attempt to relieve the constipation of strangulated hernia with cathartics. These agents add to the entanglement and increase the occlusion For the relief of the pain, opium is the great remedy, given in positive doses and preferably by the hypodermic method. Besides giving nature an opportunity to extricate the gut from the intussusception, to untwist in volvolus, and by relaxation, to overcome entanglement, preparations of opium rescue the patient from collapse, lessen inflammatory action, and preserve a condition more favorable for operative measures, which, in many cases, is the only resource for permanent relief. It must be remembered, however, that this remedy is not to be given to the extent of narcotism, which condition would mask the symptoms that are to guide the decision of important steps in the treatment.

Copious injections of warm water are of great service when carefully administered by the physician himself. Place the patient in Sims' left lateral position and administer an anæsthetic. Use very little force, work slowly, with frequent intervals of rest. If after a reasonable time—twelve, or, at most, twenty-four hours—the obstruction is not overcome by the treatment indicated, he would resort to laparotomy. He counsels the practioner not to be deterred by the statistics of laparotomy as given in standard works on surgery, for these statistics are made up of cases in which the operation was done too late. To delay the operation until death is imminent is inexcusable.—*Practitioner.*

**Hypnone.**—Von Schuder writes of fourteen patients treated by acetophenone, in whom favorable results followed. A dose of from 2 to 4 drops was sufficient to produce sleep of several hours' duration; the effect was especially happy among the phthisical.

No ill after-effects were observed. In one case only, after 6 drops had been given, the patient awoke from a long sleep with headache and slight vomiting. The effect, dependent on the dose and the individual peculiarities of each patient, was manifested after from one-half to one and one-half hours.—*Der Pharmaceut.*, February 1, 1887.

**Sarracenia Flava in Sporadic Dysentery.**—In the year of 1879 Miss B., age forty, and born of healthy parents, was taken with vomiting, cramps over the abdominal region, followed by looseness of the bowels. tenesmus, etc., and a sinking feeling. She was given by her mother the domestic remedies, such as paregoric, etc., but all to no purpose. A physician was then sent for. He prescribed, but secured only temporary relief. The following summer (1880) the same symptoms supervened, and continued at intervals until the winter set in, when they left her only to recur again. However, in the following year, when she applied to me for assistance, fearing this time that she might die of the exhaustion produced by the discharge from the bowels, I prescribed:

℞. Ext. saraceniæ flavæ (P., D. & Co.)..............℥ ij.

Sig.—Ten drops every four hours.

As she was completely run down, I prescribed the following tonic mixture:

| | | |
|---|---|---|
| ℞. | Ferri et quiniæ sulphatis........................ | ʒ iv, |
| | Tr. cardamomi comp............................ | ℥ iv, |
| | Syrup simplicis.................................. | ℥ j, |
| | Aquæ........................................... | ℥ iv, |

Mft. Sig.—A teaspoonful three times a day before meals. I did not see her again until the 30th of August, when she reported that after the first five days the stools were reduced from ten to two in number each day, and that at the time of her visit they had become natural. (I will here state that during the height of her attack she frequently had as many as thirty evacuations a day). I was both surprised and gratified at the prompt and permanent effect, for it was the first case in which I had given the sarracenia flava. The patient is now happy in the fact that for the first time since the summer of 1878 she had passed a season free from this debilitating ailment.—*Dr. Stiles in Therapeutic Gazette.*

**Berberis Aquifolium, Rhus Aromatica.**—Dr. Rees, Des Moines, Iowa, says: I have had a very considerable experience in the use of berberis aquifolium in the treatment of the secondary and tertiary manifestations of syphilis, in salt rheum, and in scaly affections of the skin, and as a result am able to report that it is a very valuable drug in these affections. So great has been my success in the treatment of syphillis, that I confidently expect to cure each new case when undertaking its treatment. It, however, requires patience and perseverance in some cases, but by exercising both, success is almost certain. I give from 15 to 20 drops in syrup and water three times a day.

As a remedy in threatened abortion, viburnum prunifolium comes well nigh being a specific. It is one of the drugs which the physician cannot afford to do without. In one case in which I employed it, the woman had had seven miscarriages at the seventh month. She consulted me on her eighth pregnancy, and under the use of viburnum prunifolium she was carried through to full term and delivered of a fine boy.

Rhus aromatica is a reliable remedy, in my experience, in incontinence of urine in children, and in one case of diabetis insipidus, it worked prompt relief.—*Therapeutic Gazette.*

**Cocaine Inebriety.**—Dr. T. D. Crothers, concludes a paper in his *Quarterly Journal of Inebriety* as follows:

The use of cocaine to excess in persons who have never used alcohol other narcotic drugs before, is very rare. Among inebriates and drug maniacs, cocaine inebriety is no doubt increasing. Its peculiar dangerous effects on the body will prevent its general use as an intoxicant to any great extent. It acts more rapidly than opium, but its effects pass off more quickly. Its first effect is more exhilarant than alcohol, but it is uncertain and variable. This stimulant action developes mania, followed by narcotism and melancholia. When given in cases of melancholia in large doses, it changes the case to mania, then finally relapses bringing back the case to melancholia again. As an intoxicant it is more dangerous than alcohol or opium. As a form of inebriety it is more difficult to treat, requiriug a longer time to break up, because of the physical complications. It cannot be used as a substitute for any other narcotic, or as an antidote or remedy.—*Practice.*

**Phosphorus in Intermittent Fever.**—A Russian provincial practitioner, Dr. Sochinski, states that during a rural practice of five years he has found phosphorus a valuable remedy in intermittent fever. He prescribes the oleum phosphoratum in the form of emulsion, a drachm in six ounces, of which a tablespoonful is given three times a day. This, he says, never produces unpleasant effects, and is, in his experience, superior to tincture of eucalyptus, and even to arsenic, cinchonine, and resorcine, though scarcely equal to quinine or carbolic acid and corrosive sublimate. It is, however, a simple and a very convenient remedy in dispensary or parochial practice.—*Medical and Surgical Reporter.*

**Iodide of Potassium in Diphtheria.**—Stepp (*Dtsch. Med. Woch.*) thinks this drug a specific in diphtheria, because of the thoroughness with which it is absorbed into the blood, whereby it "renders the bacteria innocuous." To children under three years of age he gives a teaspoonful every hour, of a two or four per cent. solution to which a small amount of tincture of iodine has been added. With older patients he uses solutions of from four to ten per cent. [It is not altogether unlikely that any good effect produced by this treatment is due to the topical action of the "small amount of tincture of iodine."]—*N. Y. Med. Journal.*

**Belladonna in Sterility of Females.**—I suppose it has fallen to the lot of almost every practitioner to be consulted by married women who never were pregnant as to the cause of their barrenness. Apparently they enjoy the best of health, and have never suffered from any irregularity of the sexual apparatus. To such I have, on several occasions, prescribed belladonna internally, and have found that, after taking the medicine for some weeks, they became pregnant. I have seen this happen so often that I am constrained to regard the occurrences as something more than accidental. I shall not venture to theorize upon its action, but will merely mention that I have observed that the external genitalia become more relaxed, and the os and cervix uteri somewhat softened and pliable, during the treatment.—*J. Harris Jones, in New York Medical Journal.*

**Pernicious Hemorrhagic Malarial Fever.**—In *Gaillard's Medical Journal*, Van Horn gives the following treatment for pernicious hemorrhagic malarial fever: A mercurial purge; quinine at once in doses of 10 to 20 grains every three hours until cinchonism is produced; then in 8-grain doses every four hours. For vomiting, a hypodermic of morphine. Bitartrate of potassium to assist the calomel and act as a diuretic and diaphoretic. Ten hours after the initial chill, wrap the patient in a blanket and put hot bricks around the body. If the chill threaten a return, give chloroform internally or morphine and atropine hypodermically. For hyperpyrexia, give quinine or antipyrin as a diuretic and hemostatic; he gives:

| | | |
|---|---|---|
| ℞. | Ext. buchu fl............................... | f ʒ iv, |
| | Ext. ergotæ................................ | f ʒ ij, |
| | Potas. acetat.................................... | ʒ jss |
| | Aquæ. q. s. ad.............................. | f ℥ vj. |

M. S.—F ℥ j every two hours until the urine clears up; then, less frequently. Turpentine may be applied over the kidneys. Pilocarpine or morphine for uremia, or chloral and bromide of potassium. For cardiac failure, whisky, or else ether or atropine hypodermically. The strength should be sustained by concentrated food.—*Medical World.*

**Posology and Use of Some New Remedies.**—*Osmic Acid*—Best administered in pill form (made up with Armenian bole). The dose is 1-60 grain, which may be repeated several times a day. Used in epilepsy and sciatica.

*Agaricine*—Best administered in combination with Dover's powder. Dose, 1-12 to 1-6 grain. Used for night sweats.

*Aloin*—From ⅛ of a grain to 3½ grains, in pill form.

*Bismuth salicylate*—Dose, from 5 to 7 grains, in pill form. In typhoid this dose may be doubled, and repeated every hour, up to ten or twelve times.

*Canabinone*—From ⅔ to 1½ grains. Best administered mixed with finely ground roasted coffee. Sedative and hypnotic.

*Euonymin*—Best given in pill form, combined with extract of belladonna or hyoscyamus. Dose, from 3 to 10 grains. Antibilious and laxative.

*Nitroglycerin* is best given in alcoholic solution. The dose is from 1-150 to 1-60 grain, repeated several times a day. Rossbach prefers ether as a solvent. His formula for its use is as follows: Dissolve 1½ grain of nitroglycerin in sufficient ether, and add the solution to a mixture consisting of 2 ounces of powdered chocolate and 1 ounce of powdered gum Arabic. Mix very thoroughly and divide into 200 pastilles. Each pastille will thus contain 1-333 grain of nitroglycerin. Used in anguina pectoris, and as a diuretic.

*Picrotoxia*—In aqueous solution. Dose, from ⅛ to ⅙ grain. Used in epilepsy.

*Liquor Magnesii Bromide*—Under this name an aqueous solution of magnesium bromide has originated and been employed in the Philadelphia Hospital, more particularly in the insane department of that institution, with such success as to warrant its more general trial and employment. Drs. Henry DaCosta and Brooke, of this city, have each employed it in insane practice, and found it to be a decided sedative in its acti n upon the nervous system, having no unpleasant after-effects, and evincing, in a number of instances, a carthartic action. As the subject may be a matter of pharmaceutical interest and inquiry in the future, I desire to communicate to you the formula I originated, which is as follows :

℞. Take of acid hydrobromic, dilute (U. S. P., 1880), pint; magnes carbonate to neutralize, or about 1 troy ounce. Filter.

Each teaspoonful contains 7 (exactly, 6.97) grains of anhydrous magnesium bromide. Dose, 1 to 2 fluidrachms.—American Journal Pharmacy, November, 1886. Joseph W. England, Ph. G.

*Osmic Acid* (Os O4) or the tetroxide of osmium is a volatile, very odorous, crystalline compound, produced by the action of nitro-hydrochloric acid on osmium or either of its lower oxides. Its vapor is very pungent and poisonous. Claus recommends cautious inhalations of sulphuretted hydrogen as an antidote. In medicine it is used in a 1-per cent. solution injected subcutaneously. Neuber recommends it against peripheral neuralgias. Mohr extols it highly in ischias rheumatica. Szumann and Eulenberg recommended parenchymatous injections in goitre, and Delbastille injects it in sarcomata and lymphomata.—*Technics.*

**Ague Treated by Quinine and Opium.**—Surgeon-Major Warburton (*Practitioner*, Nov.) says that in cases of ague, especially of the quartan form, where quinine and arsenic, either separate or combined, exert little or no influence, he has found a grain or two of opium with ten or fifteen grains of quinine exhibited an hour or two before the expected paroxysm to act like a charm. If possible the patient should go to bed in a darkened room where he falls asleep or dozes, and the attack either does not come at all or is greatly lessened in severity. The opium acting as a sedative on the vaso-motor system would thus appear to allow the quinine to exert its anti-periodic power unopposed.—*Epitome.*

**In various** affections of the eye, Juquer of Paris uses iodol, on account of its emolient qualities. It aids the cicatrization of wounds, just as iodoform does, but is without the irritating properties and disagreeable odor peculiar to the latter. He found iodol especially useful in the treatment of impetiginous eczema of the eyelids; in ulcerations of the palpebral borders and of the external canthus; in phlyctenular or pustular conjunctivitis; and also in ulcerations of the cornea, whether primary or secondary ; in chronic affections of the lachrymal ducts; in suppurative dacrocystitis; and in periosteal affections.—*Popular Science News.*

**Photographing the Uterine Cavity.**—According to the *Lancet* for May 15, 1886, a Swiss physician describes a plan of introducing wadding tampons and laminaria tents into the uterus, by which he has succeeded in dilating the organ to such an extent as to be able, by means of reflectors, to get a complete view of the whole cavity, in cases of carcinoma, fibrous polypi, fibromata and endometritis. Not content with ocular inspection, he has also contrived to obtain photographs of the cavity.—*Pacific Medical Record.*

**Thapsia Plaster.**—This plaster is made from the Thapsia garganica, which grows in abundance on the plateaus of Algeria, and since its introduction into therapeutics has rendered important service in the art of healing. It owes its irritating properties to a peculiar resin contained in its roots, rendering it a powerful derivative, whose action, on account of its form and consistence, may be graduated at will and limited to the very spot on which the physician wishes to obtain the effect. This result is impossible with croton oil, which on accunt of its fluid state spreads and acts on a larger space than desirable. From the simplicity of its use it has gained much favor with the profession, and is used by many in preference to croton oil or tartar emetic ointment, over which it has great advantages. Its revulsive action manifests itself by a miliary eruption more or less abundant according to the time it has been applied. When the eruption disappears in a few days, the plaster may be re-applied, and thus a counter-irritation kept up for any desirable length of time.—*Pacific Medical Journal.*

[The plaster will usually irritate sufficiently in ten to twelve hours and the eruption will continue three or four days. ED. RECORD.

**Elixir Iron Pyrophosphate, Quinine and Strychnine.**—Dr. C. R. Bechmann, of Fountain City, Wisconsin, says that the following formula will not precipitate by long standing:

℞. Quinine sulphate........................gr. cxx,
Strychnine sulphate........................gr. iij,
Iron pyrophosphate........................gr. ccxl,
Sodium or ammonium citrate........................gr. lx,
Alcohol........................fl ℥ ii,
Water........................fl ℥ j,
Glycerin........................fl ℥ iij,
Simple elixir enough to make fl ℥ xv.—*Digest.*

**Linseed Oil as a Remedy for Itching.**—A correspondent of the *Boston Medical and Surgical Journal* recommends the inunction of linseed oil as the best of all remedies for arresting the distressing itching of the anus with which so many persons are afflicted.—*North Carolina Medical Journal.*

**Toothache Drops.**—A solution is recommended in *L'Union Medicale*, composed of camphor, balsam of Peru and alcoholic extract of opium, of each 1 gm., and mastic 2 gm. in chloroform 20 gm. A pellet of cotton moistened with this liquid is introduced into the cavity of the tooth.

